# Predictors of substance use among Jimma University instructors, Southwest Ethiopia

**DOI:** 10.1186/s13011-019-0248-8

**Published:** 2020-01-08

**Authors:** Abraham Tamirat Gizaw, Demuma Amdisa, Yohannes Kebede Lemu

**Affiliations:** Institute of Health, Faculty of Public Health, Department of Health, Behavior, and society, Jimma University, Jimma, Ethiopia

**Keywords:** Predictors, Substance use, Substance use disorder, Jimma University

## Abstract

**Background:**

Use of substances such as alcohol, khat leaves (*Catha edulis*) and tobacco has become one of the rising major public health and socioeconomic problems worldwide and dramatically increased in developing countries. The aim of this study was to assess the predictors of substance use among Jimma University instructors.

**Method:**

Institutional based cross-sectional study design was conducted in 2018 among Jimma University instructors. A two-stage cluster sampling procedure was employed to select study participants by their departments and data was collected using structured, self-administered questionnaire with severity assessed by the standardized fifth version of a diagnostic statistical manual of mental health criteria for substance use disorder. Multivariate logistic regression was used to identify independent predictors of substance use. Variables with a *P*-value < 0.05 in the final fitting model were declared to be associated with the outcome variable.

**Results:**

A total of 330 instructors were involved in this study, with a response rate of 96.2%. About 225 of the respondents have ever used the substance in life (khat, alcohol, or cigarette or all) making the lifetime prevalence of substance use 68.2%. The lifetime prevalence of khat chewing, alcohol use, and smoking cigarette was 51.6, 81.3, and 17.3% respectively. The prevalence of substance uses disorder among users was 36.9%. Living with family (AOR = 0.220 [2.004–8.536] 95%CI), no family substance use history (AOR = 0.220 [0.098–0.495] 95% CI), friends substance use (AOR = 9.047 [4.645–17.620] 95% CI), Social norm favors substance use, (AOR = 1.123 [1.020–1.238] 95% CI), perceived benefit of substance use (AOR = 1.077 [1.008–1.151] 95% CI) were predictors of substance use.

**Conclusion:**

Perception toward substance, the influence of family and peer were associated with substance use. Therefore, designing a multifaceted approach directed to an individual, interpersonal and community-level intervention targeted to substance misperception and social norms contributing to substance use.

## Background

A psychoactive substance is a chemical that acts primarily upon the central nervous system when taken, and alters brain function, resulting in temporary changes in perception, mood, consciousness, and behavior [1]. Use of substances such as alcohol, khat leaves (*Catha edulis*) and tobacco has become one of the rising major public health and socioeconomic problems worldwide [2]. It is estimated that 90% of the global population aged 12 or older are classified with dependency on psychoactive substances [3]. About 230 million people or 5% of the world’s adult population, are estimated to have used an illegal drug at least once in their life. Alcohol and other drugs (Khat and tobacco) users are estimated to be 27 million, which is 0.6% of the world adult population [4].

Globally, the harmful use of alcohol causes approximately 3.3 million deaths every year (or 5.9% of all deaths), and 5.1% of the global burden of disease is attributable to alcohol consumption. Annually, 320,000 young people aged 15–29 years die from alcohol-related causes resulting in 9% of all deaths in that age group globally [4]. Generally, alcohol and drug use disorders are more common among males than females [5]. Research has shown, particularly in developing countries, has dramatically increased [6]. At least 15.3 million people have substance use disorders worldwide [7]. Substance use is often initiated in adolescence, but it is during adulthood that prevalence rates for its disorder peak [8].

Substance use is harmful leading to, decreased academic performance, increased risk of HIV and other sexually transmitted diseases, psychiatric disorders such as depression, lethargy, hopelessness, and insomnia [9]. It also undermines economic, and social development contributes to crime, instability, and insecurity. Not only that; alcohol and drug abuse is a major burden to society; causing economic costs, health cost, crime-related costs and losses in productivity [10]. Heavy consumption of alcohol, when shared with chewing khat, is associated with aggravating the situation, suicide attempts are one of it [8]. Alcohol and other drug use also cost to society, with estimated annual expenses of $185 billion in the United States for alcohol and $181 billion for other drug use and consequences [9].

In Sub-Sahara Africa, psychoactive substance use has dramatically increased in recent years. The rapid economic, social, and cultural transitions that most countries in sub-Saharan Africa is now experiencing have created a favorable condition for increased and socially disruptive use of drugs and alcohol [2]. According to the study done in Tanzania shows that a large percentage of the adults had used tobacco over the past 30 days (24.0% for Dar es Salaam and 38.8% for the old stone town in Zanzibar). Of the various kinds of tobacco, cigarettes were the most popular. For alcohol, 33.7% of the adult respondents in Dar es Salaam and 19.4% in Zanzibar had consumed alcohol over the past 30 days, with beer being the most popular drink [11]. Khat use is another psychoactive substance that is common in East Africa, the Arabian Peninsula and immigrants living in the west of these countries. In Ethiopia, the national level prevalence of khat use was estimated at 15%. The highest prevalence (64.9%) was observed from the southwestern part of Ethiopia and the lowest in 4 and 7.8% from the northern part. These studies indicated that Khat use was mainly associated with Muslim religion followers, males, alcohol drinkers and cigarette smokers [12].

In Ethiopia, alcohol and other drugs like khat are commonly used in both urban and rural areas, especially by youngsters. Khat chewing, drinking alcohol and using drugs are taken as means of spending spare time and entertainment. Khat and alcoholic drinks have been used traditionally for a long period of time, now khat is consumed through many faiths, social level and age groups [13]. According to the Ethiopian Demographic and Health Survey (EDHS), 2016 report 35% of women and about half of men (46%) reported drinking alcohol at some point in their lives. Regarding cigarette smoking and the use of any type of tobacco are rare among women (less than 1%). Four percent of men smoke any type of tobacco, among whom almost all smoke cigarettes [14].

In Ethiopian universities, different independent and fragmented studies have been conducted to assess the prevalence and predictors of khat chewing. In addition to prevalence, socio-demographic (being male gender), and other predictors like peer pressure, family khat chewing practice, alcohol drinking, and cigarette smoking were the most common predictors reported by the studies [15]. Smoking cigarettes, drinking alcohol, and chewing khat were widely prevalent among men. Among men, the prevalence of current daily smoking was 11.0%. Binge drinking of alcohol was reported by 10.4% of men. Similarly, 15.9% of men regularly chewed khat. Consequently, 26.6% of men and 2.4% of women reported practicing one or more of the behaviors [16]. A similar study was done in Mekele university Ethiopia showed that 82 % of ever users of sleeping pills were current users; nearly 72% ever khat users were currently chewed khat, and approximately 67% ever smokers were persisted to smoke currently. Comparably, 65% of cannabis ever users have consumed 30 days prior to the study. Heroin 10 and cocaine 14 were the least current consumed drugs [17].

In Ethiopian University not only students but also instructors use the psychoactive substance. The main reason given for smoking among university instructor is, for relaxation with friends (47.1% of ever smokers) followed by peer pressure (23.5%) and to keep alert while reading as well as for relaxation with friends was the main reason for starting chewing 40, 31.7% respectively [18]. Another study done on instructors and students showed that the prevalence of khat in Ethiopia has been reported as 32 and 42% respectively. Khat chewing is believed to affect a large segment of the Ethiopian population, especially the productive age group. It has a negative impact on health, socio-economic and political matters [19]. Prolonged and excessive use of khat is linked with several health problems [20]. A study done in Jimma town on prisoners related to substance use disorder shows that the overall prevalence of substance use disorder was 55.9%. The prevalence of khat abuse was 41.9%; alcohol use disorder, 36.2%; nicotine dependence, 19.8%; and cannabis use disorder, 3.6% and a family history of substance use were positively associated with substance use disorder [21].

Substances consumption is not legally prohibited in Ethiopia except for tobacco smoking in public places. Culturally substance is consumed in social gatherings and among friends as a leisure time activity and relaxation experience. Besides this, alcohol production like beers is increasing with a huge irresponsible advertisement.

Even if substance use has become a common problem in Ethiopia, most of the studies done mainly focused on adolescents and university students. Contrary, there is a scarcity of information available regarding the problem among adults. Moreover, university instructors are a segment of a population who can contribute a great role in the prevention of initiation of substance use among university students and the backbone for the development of a country as well. So, an assessment of substance use and associated factors is important to help efforts in reducing undesired consequences of it. Therefore the aim of this study is to assess predictors of substance use among university instructors at Jimma University, Ethiopia.

## Method and materials

### Study design and setting

The institution-based cross-sectional study design was conducted from 19th March to 20th May 2018 at Jimma University, Ethiopia. Jimma University is located in Jimma city, Oromia regional state, 335 km southwest of Addis Ababa. There are four campuses in the University (Main campus, Technology campus, college of Business and Economics, and Agricultural campus) with a total of 1687 teaching staff within nine colleges.

### Sample size

The sample size was determined using a single population proportion formula with the assumption of 95% confidence level, 5% marginal error, 10% non-response rate and the (p), the proportion of substance use taken to be 50%.

The sample size was determined using formula (n):

$$ n=\frac{{\left({Z}_{\alpha /2}\right)}^2(p.q)}{d^2} $$

$$ n=384 $$

Since the source population is less than 10, 000, using population correction formula, NF = n/1 + n/N, where, N; Source population all teaching staff of Jimma University in 2017/2018 = 1687 NF is; required sample size, and n; calculated sample size = 384. The total sample size was 312. By considering a 10% non-response rate, the final sample size was 343.

### Sampling procedure

**Fig. 1:**
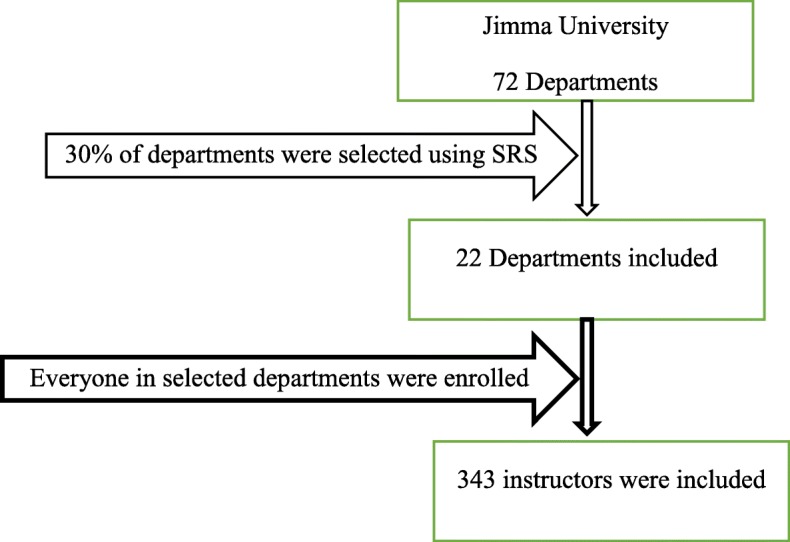
Sampling procedure for predictors of substance use among Jimma University instructors, 2018

A two-stage cluster sampling procedure was employed to select study participants from Jimma University (JU) academic staff. First, 30% of study departments were selected from the total department found on the campus. Then, each academic staff under each selected department was included in the study. Computer-generated random numbers were used to select the department based on lists of the department (Fig. 1).

### Data collection tools

Data were collected using a structured questionnaire adapted from different studies and modified accordingly. The questionnaire was translated into the local language, Amharic and then back-translated to English by language experts. The questionnaire was structured into five sections: (a) socio-demographic data (b) substance-related perceptions (c) social influence (d) precipitators for substance use (life stressors and depression level) were assessed as potential predictive factors for substance use.

The severity of the outcome variable (substance use) disorder level assessed by using the latest version of the fifth diagnostic statics manual of mental health, (DSM-5) criteria for substance use disorder. The DSM-5; a diagnostic criterion of substance use disorder is, simplified and characterized by severity rather than distinctions between abuse and dependence. It is a standardized tool, which works for all countries around the world. The reliability coefficient of the tool for this study was 0.974. Individuals with two positive answers were taken under substance use disorder.

### Data quality control

The questionnaire was prepared in English and pretested on (5%) of the sample, at the nearby university instructors, Mizan-Tepi University, which is 298 km away from Jimma town. Seven data collectors and two facilitators were trained, and proper instruction was given by the investigator before the survey. The collected data reviewed and checked on a daily base for completeness before data entry.

### Measurements and operational definitions

**Substance**: three commonly used psychoactive drugs: alcohol, cigarette, and khat.

**Substance Use**: Taking any of the three commonly used psychoactive substances: alcohol, cigarette and/or khat.

**Substance use disorder**: From the eleven DSM criteria questions, if respondents who used any of the three substances answer yes for two questions.

**Lifetime prevalence**: the proportion of individuals who had ever used the substance in their lifetime.

**The current prevalence of substance use:** the proportion of individuals who used substances within one month preceding the study.

**The perceived benefit of substance**; the summed score of six items of Likert scale approaching to a maximum sum score considered to a high perceived substance benefit.

**Perceived risk of substance**: the summed score of six items of Likert scale approaching to a maximum sum score considered a high perceived risk of substance use.

**Family support function**: the summed score of nine items of Likert scale approaching a maximum sum score considered a high family support function.

**Social norm**: the summed score of four items of Likert scale approaching a maximum sum score considered important individuals or groups that approve the respondent’s substance use.

**Perceived availability of substance**: summed score of three items of Likert scale approaching to maximum sum score considered a high perceived availability of substances.

**Perceived accessibility of substance**: the summed score of three items of Likert scale approaching a maximum sum score considered high perceived accessibility of substance.

### Statistical data analysis

Data were coded and entered using Epi Data version 3.1 then exported to statistical package for social science (SPSS) version 20 for analysis. After cleaning data, descriptive statistics such as frequency, proportions, and percentage were done for categorical variables while the measure of central tendency and dispersion conducted for numerical variables. Logistic regression analysis was used to identify factors associated with substance use. Bivariate logistic regression carried out to select a candidate for multivariable logistic regression analysis with *P*-value < 0.25 then, the candidate variables were entered into multiple logistic regressions using a backward method to identify statistically significant predictors of substance use and to control the possible confounders. The degree of association between independent and dependent variables assessed using odds ratio and statistically significant factors were declared at 95% confidence interval at a *P*-value of less than 0.05. Finally, the test for model fitness was done using the Hosmer-Lemeshow model test. The multicollinearity of the independent variable was checked by the variance inflation factor (VIF).

## Result

### Socio-demographic characteristics

A total of 330 instructors were involved in this study, making a response rate of 96.2%. Out of the total respondents, 276(83.9%) were male and, 146(44.4%) found in the age group 25–29 years. The mean age was 29.73 ± 6.46 (SD). More than half of the respondents were single 187(56.8%), and 164(49.8%) living alone as current living arrangements. The majority of the respondents of the study, 197 (59.9%) were second-degree holders and at the college of health science 105(31.9%). The mean incomes of the respondents were 5650.05 Birr (SD 1748.554) (See Table 1).

**Table 1:** Socio-demographic characteristics of instructors, Jimma University, Southwest Ethiopia, 2018

Variables		Frequency	Percent (%)	Substance use	
				Yes n (%)	No n (%)
Sex	Male	276	83.9	197 (87.6%)	79 (76.0%)
	Female	53	16.1	28 (12.4%)	25 (24.0%)
Age	<=24	56	17.0	27 (12.0%)	29 (27.9%)
	25–29	146	44.4	99 (44.0%)	47 (45.2%)
	30–34	72	21.9	59 (26.2%)	13 (12.5%)
	35–39	24	7.3	15 (6.7%)	9 (8.7%)
	40–44	18	5.5	14 (6.2%)	4 (3.8%)
	> = 45	13	4.0	11 (4.9%)	2 (1.9%)
Marital status	Single	187	56.8	121 (53.8%)	66 (63.5%)
	Ever Married	142	43.1	104 (46.2%)	38 (36.5%)
Living arrangement	Live alone	164	49.8	101 (44.9%)	63 (60.6%)
	Live with friends	39	11.9	26 (11.6%)	13 (12.5%)
	Live with family	126	38.3	98 (43.6%)	28 (26.9%)
Educational status	Diploma	9	2.7	6 (2.7%)	3 (2.9%)
	First degree	102	31.0	61 (27.1%)	41 (39.4%)
	Second degree	197	59.9	142 (63.1%)	55 (52.9%)
	Third degree and above	21	6.4	16 (7.1%)	5 (4.8%)
Religion	Orthodox	169	51.4	133 (59.1%)	36 (34.6%)
	Muslim	65	19.8	50 (22.2%)	15 (14.4%)
	Protestant	81	24.6	31 (13.8%)	50 (48.1%)
	Catholic	2	.6	2 (0.9%)	0 (0.0%)
	Others	12	3.6	9 (4.0%)	3 (2.9%)
Frequency of visiting worshiping place	Never	32	9.7	16 (7.1%)	16 (15.4%)
	A few times a year	81	24.6	57 (25.3%)	24 (23.1%)
	Once or twice a month	48	14.6	37 (16.4%)	11 (10.6%)
	Every week	109	33.1	72 (32.0%)	37 (35.6%)
	Every day	59	17.9	43 (19.1%)	16 (15.4%)
Childhood residence	Rural	94	28.6	57 (25.3%)	37 (35.6%)
	Small town	146	44.4	105 (46.7%)	41 (39.4%)
	Urban	89	27.1	63 (28.0%)	26 (25.0%)

### Substance-related perception

The respondent’s mean score for risk perception of substance use was 21.85 ± 8.23 (SD); while the perceived benefit of substance use 13 ± 7.11 (SD). Whereas, the mean score of the respondent’s perceived availability and perceived accessibility of substance were 8.85 ± 4.02 (SD), 7.48 ± 3.87 respectively. The mean score for the perceived affordability of substance was 2.95 ± 1.57 (SD).

### Social influences

One hundred twenty-six (38.18%) of the respondents had a family history of substance use. Whereas for those who currently use substances the number of family substance usage accounted were almost equal to those whose families were not used, 113(50.2%) and 112(49.8%) respectively. The mean score for the family support function of the respondent was 37.29 ± 7.15 (SD). Regarding respondent’s friends’ substance use behavior; 196(87.1%) of the respondents had friends who had used the substance. Respondents’ mean score for social norm accounted for 8.93 ± 4.97 (SD).

### Substance use precipitators

Respondents’ mean score for life stressors were 24.45 ± 11.08 (SD). While the mean score of the respondent’s depression status was 17.12 ± 7.71 (SD). Reason for using substance; among respondents who chewed khat 62% was for reading and 54.6% for liking the feeling. Those instructors who drank alcohol 41% were for getting relief from sadness and like the feeling.

### Prevalence of substance use

Regarding the history of substance use, 225 (68.2%) of the respondents had ever used the substance. Among total respondents, 120 (53.3%) chewed khat in their lifetime and almost all of them chewed in the past thirty days 117 (97.5%). Of them, about 46(38.3%) respondents chew at least once in a week. From those who had a history of substance use; the majority of them 183(81.3%) used alcohol in their life, as well as drunk in the past thirty days 223 (99.1). Frequency of drinking lead by at least once in a month 89(48.6%), and the number of users decreased as the frequency of usage increased from at least once in a week 67(36.6%) to daily 4(2.2%). From the types of drinks containing alcohol, majority of the respondents frequently drank beer 150 (82.0%) with mostly drinking one-two bottles at particular day 86 (47.0%) and, the number of users decreased as the dose/bottle increased to three to four, five to six, and seven to nine; 58 (31.7%), 32 (17.5%), 7 (3.8%) respectively. From substance users, 39 (17.3%) respondents have smoked cigarettes in life and all of them (100%) smoked in the past thirty days. Of the respondents smoked cigarette 14(35.0%) were on a daily bases, and less than five cigarettes per day 26(66.7%). From those who used substance 141(62.7%) of them used only one of the substances from the three, while 55(24.4%) and 29(12.9%) of them used two and all of the three substances respectively. The most commonly used substance among instructors was alcohol followed by khat and cigarettes (See Table 2).

**Table 2:** Substance use characteristics of instructors in Jimma University, Southwest Ethiopia, 2018

Variable	Frequency(*n* = 330)	Percent
Ever used Substance in life		
Yes	225	68.4
No	104	31.6
Khat use in life		
Yes	120	53.3
No	109	48.4
Khat use in the past thirty days		
Yes	117	97.5
No	3	2.5
Frequency of khat chewing		
never	3	2.5
at least once in a month	30	25.0
at least once in a week	46	38.3
more than three days in a week	31	25.8
daily	10	8.3
Alcohol use in life		
Yes	183	81.3
No	42	18.7
Alcohol use in thirty days		
Yes	223	99.1
No	2	.9
Frequency of using alcohol		
never	2	1.1
at least once in a month	89	48.6
at least once in a week	67	36.6
more than three days in a week	21	11.5
daily	4	2.2
Type of drink contain alcohol		
beer	150	82.0
wine	20	10.9
sprit	4	2.2
hard liquor vodka, whisky	4	2.2
local drinks	3	1.6
mixed drinks	2	1.1
Number of drink at particular day		
one-two	86	47.0
three-four	58	31.7
five-six	32	17.5
seven-nine	7	3.8
Cigarette smoking in life		
Yes	39	17.3
No	186	82.7
Cigarette in the past thirty days		
Yes	225	100.0
No	0	0
Frequency of smoking		
at least ones in a month	3	7.5
at least once in a week	10	25.0
more than three days in a week	13	32.5
daily	14	35.0
Number of cigarette in particular day		
less than five	26	66.7
six-ten	12	30.8
eleven-fifteen	1	2.6

### Context of use

The most preferred time of use for the respondent was; in the afternoon 95(79.2%) for chewing khat, at night 155(84.7%) for drinking alcohol, and 15(38.5%) of them any time for smoking cigarette. The majority of respondents who used substances preferred their friends both to chew khat with 75(63.6%) and, 147(80.3%) for drinking alcohol. The mean age for initiation of substance was found to be 20 ± 2.83 (SD).

#### Substance use disorder prevalence and characteristics

Of the total of 225 respondents who used the substance, 83 of them were in the substance use disorder whereas the rest 142 was not in the substance use disorder making the prevalence of substance use disorder 36.9%. Of the total 83 instructors who had substance use disorder, 48 (58%) had mild substance use disorder and 19(23%) had moderate substance use disorder. However, 16 (19%) of the instructors had severe substance use disorder which affected their daily activities and their life (See Table 3).

**Table 3:** Substance Use Disorder (SUD) using DSM-V Criteria among instructors in Jimma University, Southwest Ethiopia, 2018

Types of drugs	Current use n (%)	Life time use n (%)	Substance use disorder n(%)
Alcohol	156 (47.3)	183 (55.4)	44 (24.0)
Khat	97 (29.4)	120 (36.4)	30 (25.0)
Cigarette smoking	30 (9.1)	39 (11.8)	9 (23.1)

### Factors associated with substance use

From those candidate variables in bivariate analysis; living arrangement, family substance use history, friends substance use history, perceived benefit of substance and social norm were found to be significant predictors of substance use among instructors. Instructors who lived with families were 4 times more likely to use substances than those who live alone (AOR =4.136 [2.004–8.536] 95% CI). Instructors with no family history of substance use had 4.5 times less risk of using the substance as compared to those instructors with a family history of substance use (AOR = 0.220 [0.098–0.495] 95% CI). Meanwhile, instructors with a friend’s history of substance use had a 9 times higher risk of substance use as compared to those instructors with no friends history of substance use (AOR = 9.047 [4.645–17.620] 95% CI). As a perceived benefit of the substance of instructors increases by one unit the odds of becoming at risk for substance use increases by 1.1 (AOR =1.077 [1.008–1.151] 95% CI). As social norms to substance use increase by one unit, the odds of becoming at risk for substance use increase by 1.12 (AOR =1.123 [1.020–1.238] 95% CI) (See Table 4).

**Table 4:** Multivariable logistic regression for substance use among instructors, Jimma University, Southwest Ethiopia, 2018

Variables n = 330	Substance use		OR (95%CI)	
	Yes n (%)	No n (%)	Crude	Adjusted
Living arrangement				
Live alone	101 (44.9%)	63 (60.6%)	1	1
Live with friend	26 (11.6%)	13 (12.5%)	0.458 (0.271–0.774)	0.687 (0.223–2.112)
Live with family	98 (43.6%)	28 (26.9%)	0.571 (0.260–1.256)	4.136 (2.004–8.536)**
Friends substance use history				
Yes	196 (87.1%)	69 (66.3%)	.075(.043–.132)	9.047 (4.645–17.620)**
No	29 (12.9%)	69 (66.3%)	1	1
Family substance use history				
Yes	113 (50.2%)	12 (11.5%)	1	1
No	112 (49.8%)	92 (88.5%)	0.129 (0.067–0.249)	0.220 (0.098–0.495)**
Perceived benefit of substance			1.130 (1.083–1.179)	1.077 (1.008–1.151)**
Social norm			1.231 (1.143–1.326)	1.123 (1.020–1.238)**

**Identified as factors for multivariable logistic regression analysis (P < = 0.05)

## Discussion

This study revealed that the prevalence of substance use among Jimma University instructors was 68.4% which is consistent with the study done in Jimma zone which was 68.5% [22]. On contrary, the prevalence of substance use found in this study is relatively higher than the study done in Gondar university instructors which are 42% of the instructors were either lifetime cigarette smokers or khat chewers or both [11]. The difference may be due to the current study have alcohol use in addition to the prevalence and risk factors of cigarette smoking and khat chewing. In addition in this study substance use was taken as using any of substances like khat chewing, cigarette smoking or alcohol drinking. The geographic differences and the availability of excessive khat production in the area may be contributed to the differences.

The lifetime prevalence of alcohol drinking found in this study was 81.3% is relatively higher than that of the study done in India (33.78%), Zambia (61%), and (67%) in a rural part of South Africa [11, 23, 24]. These differences might be due to socio-cultural differences and study population size. The previous studies were conducted in the community on a large population while the current study was institution-based and conducted in small study subjects. This result for alcohol prevalence is also higher than, a cross-sectional study was done in Addis Ababa and Jimma town in the past twelve months alcohol consumption was 69 and 50% respectively [16, 22].

The lifetime prevalence of khat chewing found in this study was 53.3%, higher than the study done among Gondar University instructors 21% and in Addis Ababa adults 18.3%, while it is lower than the study done in Jimma 68.5% [16, 18, 22]. The difference might be the study settings in which khat chewing is common in Jimma than in Gondar and in Addis Ababa. Meanwhile, the result of this study was lower than that done in Jimma town since the current study was conducted in one institution instructors. The lifetime prevalence of cigarette smoking in this study was 17.3%. This was higher than the study done among Debre-Berhan University students which were 7.4% [25]. This difference might be due to the instructor’s financial capability to afford the price of cigarettes relative to the students. Whereas study was done in different countries in Africa, America and Asia showed higher lifetime prevalence which was 27.8% in Sudan [10], 26% in America, 22.84% in India,30% in South Africa,31% in Zambia, and 19.7% in Zanzibar [23–27]. The difference may be due to the socio-cultural difference between the study settings. The prevalence of tobacco results in this study is also lower than the study conducted in Jimma town 35.5% and Jimma psychiatric outpatient ward 20.5% [22, 28] and Jimma town prisoners which was 19.8% [21].

This study showed that the prevalence of substance use disorder among Jimma University instructors was 36.9%. The current finding was lower than the study done in Jimma town prisoners which were 55% [21]. This is also relatively higher than the study conducted in Ukraine and USA lifetime prevalence rates of substance use disorders 15 and 8% respectively [29, 30]. The possible reason could be due to population size differences and study settings. The previous studies were population-based surveys starting from 12 years old individuals while this study conducted only among one institution instructors. In addition, the difference might be due to the fact that these countries have better behavioral therapy services to prevent as well as an early treatment center for substance use disorder before its magnitude showed boldly.

Regarding predictive factors, this study showed that instructors who live with family were 4 times more likely to use substances than those who live alone counterparts (AOR = 4.136, [2.004–8.536] 95% CI). This might be due to the social norms and religion conditions that the family perceives some substance use as normal behavior and religiously connected; especially the khat chewing considered normal at weekends and holidays among Muslims. Similarly, alcohol consumption in the special holidays is considered normal in Orthodox Christianity followers which might be contributed to substance use with families posing them at risk of psychoactive substance use behavior. The finding is in line with other studies done in India that assessed the prevalence and the pattern of substance abuse and revealed that living alone or with a friend is factor less often associated with substance use [23].

This study showed that instructors with no family history of substance use had 4.5 times less risk of using the substance as compared to those instructors with a family history of substance use (AOR =0.220 [0.098–0.495] 95% CI). The finding of the current study was consistent with a study done in high school students in Woreta town, North East, Ethiopia [31]. It is also consistent with systematic analysis conducted to summarize the key epidemiologic literature that has studied social (or exogenous) factors that may shape substance use behavior and showed that, parental substance use appears to be the primary social factors associated with smoking and alcohol initiation [32].

The other predictive factor which revealed in this study were instructors with a friend history of substance use had a nine times higher risk of substance use as compared to those instructors with no friends history of substance use (AOR = 9.047 [4.645–17.620] [95% CI]). The current study was similar to the study done in Hawassa University students on alcohol and khat use; students who had a friend who uses the substance had 4.6 times higher odds of substance use than those students who had no friends who used substances [33]. Also, the finding of the current study was in line with another study revealed that students who had friends who used substances had 2.14 times higher risk of using substances than those students who had no friends who had used substances, even though the study population was different [31].

Another predictive factor for substance use which the study revealed was the social norms that favor substance use. As the social norm, that favor substance use increases the likelihood that instructors to use substance increases too. This study is in line with the previous study done in Ethiopia, which shows that community norms favorable to substance use were two times more likely to lead to adolescent substance use than community norms that were not favorable to substance use even though the study was conducted among the different population [31]. Similar findings were also reported from the study done among college freshman, perceived peer drinking norms were positively correlated with both alcohol consumption and alcohol problems [34].

This study revealed that the perceived benefit of using a substance is a predictor of substance use among instructors. The possible explanation could be, when instructors perceive using substance benefits, they tend to use it by taking it as a reason for its advantage. Conversely, as the instructor’s perceived benefit of using a substance is less or the perceived risk of using a substance is high the likelihood of engaging in substance use among instructors decreases [18].

## Conclusion

In the present study living arrangement, family substance use history, friend’s substance use history, social norms and perceived benefit of substance use were positively associated with substance use among instructors. The influence of family, peer, as well as society at large, plays a great role for the instructors to use substances than socio-demographic factors. So that, substance use is the result of a multiplicity of factors and cannot be corrected by a single intervention. Moreover, it should be prevented by start working from individual to the community level, in a way that the risk of substance taking is understood and the norms of the community become favorable in ensuring positive health for the individual as part of the community. Therefore designing a multifaceted approach directed to an individual, interpersonal and community-level intervention targeted to substance misperception and social norms contributing to substance use.

## Data Availability

The datasets used and analyzed during the current study are available from the corresponding author on reasonable le request.

## Abbreviations

AUD: Alcohol Use Disorder
DSM: Diagnostic statistical manual of mental health
EDHS: Ethiopian Demographic and Health Survey
HIV: Human Immune Deficiency Virus
KAP: Knowledge, Attitude, Practice
NCSR: National Comorbidity Survey Replication
SRS: Simple Random Sampling
SUD: Substance Use Disorder
WHO: World Health Organization
